# Polysomnographic nighttime features of Restless Legs Syndrome: A systematic review and meta-analysis

**DOI:** 10.3389/fneur.2022.961136

**Published:** 2022-08-25

**Authors:** Chaofan Geng, Zhenzhen Yang, Tingting Zhang, Pengfei Xu, Hongju Zhang

**Affiliations:** ^1^Henan University People's Hospital, Henan Provincial People's Hospital, Zhengzhou, China; ^2^Fuwai Central China Cardiovascular Hospital, Henan Provincial People's Hospital, Zhengzhou, China; ^3^Zhengzhou University People's Hospital, Henan Provincial People's Hospital, Zhengzhou, China

**Keywords:** Restless Legs Syndrome, Polysomnographic, meta-analysis, pathophysiology, sleep

## Abstract

**Background:**

Restless Legs Syndrome (RLS) is a common sleep disorder. Polysomnographic (PSG) studies have been used to explore the night sleep characteristics of RLS, but their relationship with RLS has not been fully analyzed and researched.

**Methods:**

We searched the Cochrane Library electronic literature, PubMed, and EMBASE databases to identify research literature comparing the differences in polysomnography between patients with RLS and healthy controls (HCs).

**Results:**

This review identified 26 studies for meta-analysis. Our research found that the rapid eye movement sleep (REM)%, sleep efficiency (SE)%, total sleep time (TST) min, and N2 were significantly decreased in patients with RLS compared with HCs, while sleep latency (SL) min, stage shifts (SS), awakenings number (AWN), wake time after sleep onset (WASO) min, N1%, rapid eye movement sleep latency (REML), and arousal index (AI) were significantly increased. Additionally, there was no significant difference among N3%, slow wave sleep (SWS)%, and apnea-hypopnea index (AHI).

**Conclusion:**

Our findings demonstrated that architecture and sleep continuity had been disturbed in patients with RLS, which further illustrates the changes in sleep structure in patients with RLS. In addition, further attention to the underlying pathophysiological mechanisms of RLS and its association with neurodegenerative diseases is needed in future studies.

## Introduction

Restless Legs Syndrome (RLS), also known as Willis-Ekbom disease (1), is a common neurological sensory-motor disorder, mainly characterized by strong discomfort of the lower limbs at night or at rest and the irresistible desire to move the legs (2). The prevalence of RLS is ~0.1–15% (3). Currently, RLS can be divided into idiopathic and secondary forms (4). Alternately, secondary RLS may be related to other medical causes, such as anemia, pregnancy chronic renal failure, and iron deficiency (5). Previous genome-wide association studies had demonstrated that MEIS1, LBXCOR1, and BTBD9 are the RLS-predisposing genes (6–8), which increase the risk of RLS (9). Although the exact pathophysiological mechanisms of RLS are unknown, there is growing research evidence that dopaminergic neurons and iron deficiency are linked with the pathogenesis of RLS (3).

Polysomnography (PSG) can distinguish between non-rapid eye movement (NREM) sleep and rapid eye movement (REM) sleep, as well as stages N1–N3 of NREM sleep, which are recognized as the gold standard for assessing the sleep amount and quality of objective sleep (10). Sleep interruptions are also useful for understanding the neurobiology of neurodegeneration, according to PSG (11). For the diagnosis of RLS, PSG gives objective evidence (10, 12). A previous imaging investigation had demonstrated that there was a negative correlation between the fractional anisotropy (FA) values in the left corticospinal tract and the number of movement arousal index (MAI) in patients with RLS, implying clinical significance (13). Although previous studies have attempted to investigate changes in the nighttime features of patients with RLS, there may be heterogeneity between studies involving clinical variables (such as disease duration, medication status, and disease type), demographic characteristics (such as sex and age), and research methods (such as PSG scoring methods and adaptation nights), so the exact differences in sleep features between healthy controls (HCs) and patients with RLS have not been fully established. In various neurological illnesses, a meta-analysis for PSG parameters has been used (10, 11, 14). Furthermore, using meta-analysis to examine changes in PSG parameters in patients with RLS can not only overcome the limitations of a single study with small sample size, but it can also be used to assess the potential influencing factors that influence changes in nighttime features *via* a subgroup analysis.

To our knowledge, there has been no meta-analysis investigation into PSG-measured sleep in RLS. To close this gap, we extensively evaluated prior case-control studies and, where applicable, performed meta-analytic approaches to determine the pooled effect sizes for variations in PSG variables between patients with RLS and HCs. We also looked into factors that could contribute to study heterogeneity.

## Methods

### Information sources and search

Before 10 September 2021, two investigators (Z.-Z.Y. and P.-F.X.) conducted a systematic review of English-language and peer-reviewed articles from PubMed, EMBASE, and the Cochrane Library databases, with no restrictions on publication type or language. Polysomnography OR sleep architecture OR sleep stages OR sleep recordings AND Restless Leg^*^ *Syndrome* were among the database search criteria. On March 2, 2022, we re-ran the literature search using the same search strategy to find newly published articles. To discover qualifying articles, the full texts of possibly relevant articles were retrieved. In addition, we searched references of primary research and reviewed articles accordingly to prevent omissions. Any discrepancies were passed on to a third reviewer. The International Prospective Register of Systematic Reviews (PROSPERO) was used to register our meta-analysis (ID: CRD42021254140) (15).

### Inclusion criteria

In our study, mainly case-control studies were included to measure the nocturnal PSG differences between HCs and patients with RLS. The inclusion criteria for studies included in our final meta-analysis were as follows: (1) the International Classification of Sleep Disorders (16) or the International RLS Study Group (IRLSSG) (17) was used to define whether the patients meet the diagnostic criteria of RLS; (2) including the HCs group; (3) these studies provide data on night-related sleep parameters in subjects, which were obtained by PSG measurements; (4) studies published in English-language and peer-reviewed journals; and (5) observational or cohort studies.

### Exclusion criteria

The exclusion criteria were as follows: (1) guidelines, case series, case reports, reviews, and letters; (2) animal studies; (3) research not associated with RLS; (4) RLS secondary to Parkinson's disease (PD), peripheral neuropathy, pregnancy, iron deficiency, renal failure, chronic kidney disease, and drug-induced factors; (5) combined with other sleep disorders (for instance obstructive sleep apnea syndrome (OSAS) and rapid eye movement sleep behavior disorder (RBD), or narcolepsy); (6) no studies have reported the nocturnal PSG data of patients with RLS and HCs; and (7) the sleep parameter data format reported in the study cannot be converted into averages and standard deviations (SDs).

### Data collection process

Data were extracted independently using a pre-designed form and completed by two investigators. Any disagreements during the data extraction process were addressed through adequate discussion and, if necessary, will be arbitrated through a third examiner. Extracted data were entered by one investigator and verified by two reviewers. Raw data were extracted from the results of the original study with strict quality control, and the relevant corresponding author of the study was contacted if necessary.

In this study, the PSG variables examined include total sleep time (TST), wake time after sleep onset (WASO), sleep efficiency (SE), and percentage of N1, N2, and N3, REM sleep, and REM latency. In the American Academy of Sleep Medicine (AASM) scoring rules, N3 represents slow wave sleep (SWS) and also replaces stage 3 and stage 4 in the R&K nomenclature (18). Thus, the data for stage 3 and stage 4 in the included studies were also extracted for estimating SWS. Additional PSG variables include the periodic limb movements index (PLMI), apnea-hypopnea index (AHI), and arousal index (AI).

Demographic characteristics were recorded for each study, such as mean age, sex ratio (male/female), duration of disease (years), and body mass index (BMI) for patients with RLS and healthy subjects. In addition, we documented the diagnostic criteria for patients with RLS, medication status (i.e., medication-naïve, medication-withdrawn, or medicated) in patients with RLS, and adaptation night.

### Quality assessment

To identify the quality of the included studies, two investigators independently used the Newcastle-Ottawa Quality Assessment Scale (NOS) (19). The NOS consists of three components, respectively: the measurement of exposure factors, the intergroup comparability of both groups, and the selection of the study population, out of a total score of 9 (20). A total NOS score of ≥7, 4–6, and ≤ 3 was defined as high, medium, and lower quality studies, respectively (21). Any disagreements that arise from the analysis process were addressed through discussion. Arbitration by a third reviewer when necessary.

### Statistical analysis

To enter and extract data, we used Excel software. Software version Review Manager 5.3 was used for the meta-analysis. The sample size, mean, and standard deviation (SD) of patients with RLS and HCs were entered to calculate the standardized mean difference (SMD) between each group of nighttime sleep features measured by PSG. The Q statistic and I-square (*I*^2^) were calculated to test the magnitude of heterogeneity and to inform on the degree of overlap between the 95% confidence intervals (*CI*s) of different studies for the global effect-size estimate of each PSG variable. When the research results of each PSG variable were *p* > 0.1 and *I*^2^ <50%, it can be assumed that there was homogeneity among the research results, and the fixed-effects model was used for analysis. Conversely, *p* ≤ 0.1 and *I*^2^ ≥ 50% indicate the presence of heterogeneity, so the random-effects model will be used for analysis. To check for the publication bias, the Egger regression method was used; when the *p* < 0.05, bias was suggested (22). If publication bias existed, to adjust the effect sizes, Duval and Tweedie's trim and fill test will be used (23). To identify potential sources of heterogeneity between studies, a subgroup analysis will be performed. The stability of the results was tested using sensitivity analysis. All significant values were set at *p* < 0.05 in this study.

## Results

### Study selection

**Figure 1:**
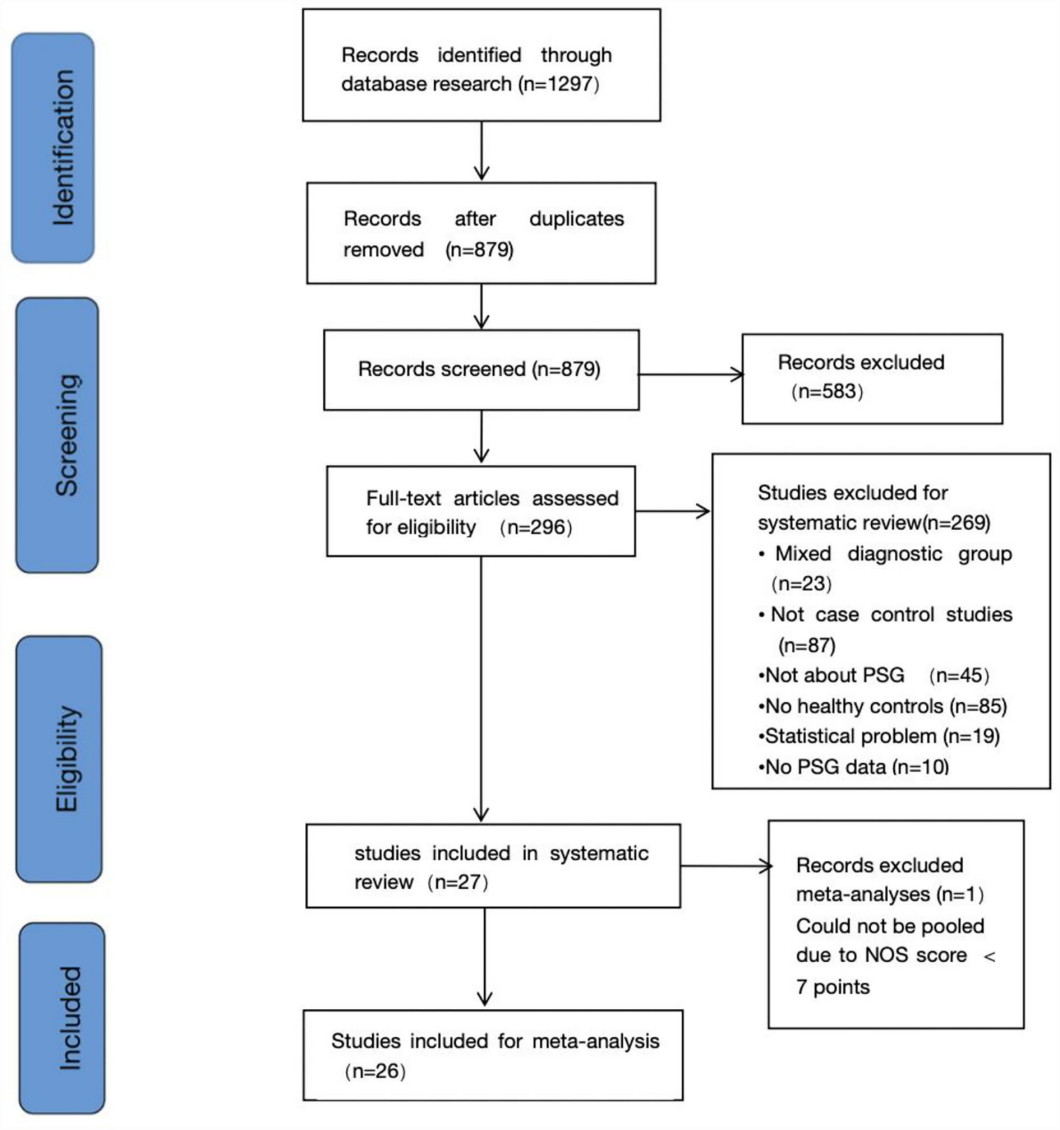
Flow diagram of the studies selection.

The search identified a total of 1,297 candidate studies, of which 418 were duplicates. In addition, 583 studies were excluded by reviewing titles and abstracts. Immediately after, 296 studies were reviewed in full text. In addition, no studies eligible for inclusion were found by searching the reference list for potentially relevant studies. Finally, we determined that 27 studies existed that met the inclusion criteria, 26 of which were included in the meta-analysis (13, 24–49). Since one of the studies had a score of <7 on the NOS, it was excluded (50). Figure 1 shows the process of study selection. Table 1 summarizes the characteristics of the included studies.

**Table 1:** Characteristics of participants included in the studies.

**Number**	**Study selection**	**Country**	**Type of study**	**Health status**	**Sample size**		**age**		**Sex ratio (male/female)**		**Medication**	**PSG night**	**Diagnostic criteria**
					**RLS**	**HC**	**RLS**	**HC**	**RLS**	**HC**			
1	Martin Michau (22)	Canada	Case-control study	General population	100	50	48.8 ± 11.5	48.4 ± 9.2	60/40	29/21	NO	1	IRLSSG
2	Diego Garcia-Borreguero (25)	Spain	Case-control study	General population	12	12	54.9 ± 11.2	53.8 ± 8.5	5/7	5/7	NO	1	IRLSSG
3	Plazzi G (26)	Italy	Case-control study	General population	17	17	42.8 ± 16.95	44.1 ± 17.25	6/11	7/10	NO	1	IRLSSG
4	Magdolna Hornyak, (27)	Germany	Case-control study	General population	20	20	50.4 ± 9.6	51.8 ± 7.7	10/10	10/10	NO	1	IRLSSG
5	Raffaele Ferri, (28)	Italy	Case-control study	General population	20	12	47.6 ± 12.01	46.7 ± 15.21	13/7	3/9	NO	1	IRLSSG
6	Gwendolyn Boehm, (29)	Germany	Case-control study	General population	95	31	54.6 ± 11.1	59.3 ± 9.4	49/46	15/16	NO	1	IRLSSG
7	Raffaele Ferri, (30)	Italy	Case-control study	General population	90	28	58.2 ± 11.84	53.1 ± 19.55	37/53	12/16	NO	1	IRLSSG
8	Kwang Su Cha, (31)	South Korea	Case-control study	General population	15	15	45.73 ± 11.78	49.00 ± 7.55	2/13	0/15	NO	1	IRLSSG
9	Jung-Ick Byun, (32)	South Korea	Case-control study	General population	4	4	53 (52–57)	52 (43–58)	0/4	0/4	NA	1	IRLSSG
10	Stefan Seidel, (33)	Austria	Case-control study	General population	7	7	55.4 ± 13.1	50.9 ± 19.4	2/5	3/4	NA	1	IRLSSG
11	Claudia Schilling, (34)	Germany	Case-control study	General population	73	34	54.8 ± 13.2	49.7 ± 8.3	27/46	11/23	NO	1	IRLSSG
12	De Cock VC, (35)	France	Case-control study	General population	25	25	64 ± 5	65 ± 5	Na	Na	NO	1	IRLSSG
13	Yves Dauvillier, (36)	France	Case-control study	General population	108	45	61.54 (24.10; 85.03)	53.91 (23.00; 74.87)	43/65	17/28	NO	1	IRLSSG
14	Wetter TC, (37)	Germany	Case-control study	General population	10	8	56 ± 6	57 ± 5	Na	Na	NO	1	IRLSSG
15	Saletu B, (38)	Austria	Case-control study	General population	12	12	57.2 ± 11.7	59.0 ± 15.9	Na	Na	NO	1	IRLSSG
16	Mariusz Sieminski, (47)	Poland	Case-control study	General population	30	27	49.0 ± 14.9	44.3 ± 16.3	12/18	12/15	NO	1	IRLSSG
17	Raffaele Ferri, (40)	Italy	Case-control study	General population	22	22	29.0 ± 8.62	30.9 ± 6.18	13/9	12/10	NO	1	IRLSSG
18	Magdolna Hornyak, (41)	Germany	Case-control study	General population	45	45	47.4 ± 10.9	47.3 ± 10.5	16/29	16/29	NO	1	IRLSSG
19	Raffaele Ferri, (42)	Italy	Case-control study	General population	34	13	57.6 ± 9.44	55.5 ± 11.88	11/23	6/13	NO	1	IRLSSG
20	Raffaele Ferri, (43)	Italy	Case-control study	General population	27	32	8.4 ± 2.16	8.0 ± 2.14	16/11	19/13	NO	1	IRLSSG
21	Raffaele Ferri, (44)	Italy	Case-control study	General population	27	14	53.6 ± 14.90	50.3 ± 15.83	15/12	7/7	NO	1	IRLSSG
22	Thireau J, (45)	France	Case-control study	General population	64	38	61.5 ± 8.8	45.0 ± 9.5	30/34	9/29	NO	1	IRLSSG
23	Richard P. Allen, (46)	America	Case-control study	General population	28	20	62.8 ± 9.9	62.6 ± 9.3	13/15	8/12	NO	1	IRLSSG
24	Hea Ree Park, (13)	Korea	Case-control study	General population	30	31	46.3 ± 13.0	44.1 ± 12.0	22/8	23/8	NO	1	IRLSSG
25	Sofiene Chenini, (48)	France	Case-control study	General population	102	73	58.91 (24.50; 77.22)	56.85 (23.46; 76.65)	63/39	45/28	NO	1	IRLSSG
26	Sofiene Chenini, (49)	France	Case-control study	General population	84	76	55.14 ± 12.32	52.17 ± 15.30	53/31	47/29	NO	1	IRLSSG

### Description of the included studies

In our study, a total of 1,101 patients with RLS and 711 HCs were included. Of the 26 studies included, the number of participate included ranged from 8 (4 patients with RLS and 4 HCs) to 175 (102 patients with RLS and 73 HCs). The mean age range of the patients with RLS and HCs included in the study was 8.4–64 years (reported in 26 studies). The percentage of female patients across studies ranged from 0 to 100% (reported in 23 studies). Of the studies that included PSG, 25 of them excluded patients with RLS who were treated with medications whose use affected sleep quality (i.e., antidepressants, benzodiazepines, and modafinil), and two studies (32, 33) did not report the medication status of patients with RLS. All the included studies performed PSG in the sleep lab. The diagnostic criteria, medication status, and PSG nights used for the analysis are presented in Table 1.

### Research quality evaluation results

To assess the quality of the included studies, we used the Newcastle-Ottawa Scale (NOS). Based on the final total score for each study, the high quality of the literature can be inferred in our study. The details of the quality assessment for each study are presented in Table 2.

**Table 2:** Quality assessment of included studies.

	**Choose**				**Comparability**	**Exposed**			**Total score**
**Study**	**Case definition**	**Representativeness**	**Control selection**	**Control definition**	**Control for important factor**	**Ascertainment of exposure**	**Same method of ascertainment for cases and controls**	**Non-response rate**	
Michau et al. (24, 52)	1	1	1	1	2	1	1	0	8
Diego Garcia-Borreguero et al. (25)	1	1	1	1	2	1	1	0	8
Plazzi et al. (26)	1	1	1	1	2	1	0	0	7
Hornyak and Feige (25)	1	1	1	1	2	1	0	0	7
Ferri (28)	1	1	1	1	2	1	0	0	7
Boehm et al. (29)	1	1	1	1	2	1	0	0	7
Ferri (30)	1	1	1	0	2	1	1	0	7
Cha et al. (31)	1	0	1	1	2	1	1	0	7
Byun et al. (32)	1	1	1	1	1	1	1	0	8
Seidel et al. (33)	1	1	1	1	1	1	1	0	8
Schilling et al. (34)	1	1	1	0	2	1	1	0	7
De Cock et al. (35)	1	1	1	1	2	1	1	0	8
Dauvillier et al. (36)	1	1	1	1	2	1	1	0	8
Wetter et al. (37)	1	1	1	1	2	1	1	0	8
Saletu et al. (38)	1	1	1	1	2	1	1	0	8
Mariusz Sieminski, (47)	1	1	1	1	2	1	1	0	8
Ferri et al. (40)	1	1	1	0	2	1	1	0	7
Hornyak et al. (41)	1	1	1	1	2	1	1	0	8
Ferri et al. (42)	1	1	1	1	2	1	0	0	7
Ferri et al. (43)	1	1	0	1	2	1	1	0	7
Ferri et al. (44)	1	1	1	1	2	1	1	0	8
Thireau et al. (45)	1	1	1	1	2	1	1	0	8
Richard P. Allen, (46)	1	1	1	1	1	1	1	0	8
Park et al. (13)	1	1	1	1	1	1	1	0	8
Chenini et al. (39, 48)	1	0	1	1	2	1	1	0	7
Chenini et al. (49)	1	0	1	1	2	1	1	0	7
Michau et al. (52)	1	1	1	1	2	1	0	0	7

### Results of individual studies

Not all of the 15 sleep parameters were measured in all of the 26 case-control studies included in our study (SWS%, REM%, REML, SE%, SL TST, SS, AWN, WASO, N1%, N2%, N3%, AHI, AI, and PLMI). The sleep parameters tested ranged from a maximum of 10 to at least 3 sleep parameters recorded. Pooled effect sizes for a specific sleep parameter were calculated by up to 26 studies (i.e., TST) and at least 3 studies (i.e., REMI). The sleep parameters included in the different studies all have the same definition and units.

### Comparison of RLS patients and healthy controls

Some sleep parameters were transformed and unified before the statistical analysis. We conducted a meta-analysis of a total of 15 sleep parameters, TST, WASO, SE%, SL, SS, AWN, N1%, N2%, N3%, SWS%, REM%, REML, AHI, AI, and PLMI, which were included in 26, 13, 26, 16, 4, 3, 22, 23, 4, 20, 17, 3, 6, 10, and 21 studies, respectively. Regarding the macroscopic structure of sleep, meta-analysis showed that TST min [SMD = −0.37, 95% *CI*: (−0.56, −0.18)], SE% [SMD = −0.61, 95% *CI*: (−0.80, 0.41)], N2% [SMD = −0.49, 95% *CI*: (−0.62, 0.36)], and REM% [SMD = −0.31, 95% *CI*: (−0.45, −0.16)] were significantly decreased in patients with RLS compared with the HC group, and the difference was statistically significant, while the WASO min [SMD = 0.57, 95% CI: (0.11, 1.02)], SL min [SMD = 0.34, 95% *CI*:(0.18 0.50)], SS event/h [SMD = 0.64, 95% *CI*: (0.33, 0.96)], AWN event/h [SMD = 0.72, 95% *CI*: (0.33, 1.12)], N1% [SMD = 0.37, 95% *CI*: (0.15, 0.59)], REML min [SMD = 0.78, 95% *CI*: (0.39, 1.16)], AI event/h [SMD = 0.61, 95% *CI*: (0.26, 0.95)], and PLMI event/h [SMD = 1.01, 95% *CI*: (0.80, 1.23)] were significantly increased, and the difference was statistically significant. There was no significant difference in N3%, SWS%, and AHI index between the RLS and HC groups (*p* > 0.05). The pooled effect sizes for 15 sleep parameters are displayed in Table 3.

**Table 3:** Summary of meta-analysis comparing patients with Restless Legs Syndrome (RLS) patients and healthy controls (HCs).

	**No. of comparisons**	**Number of RLS samples**	**Number of HC samples**	**SMD (95% CI)**	**P Value**	**Effect model**	**Heterogeneity**			
							**Q Statistic**	**df**	**I^2^ Statistic**	**P Value**
TST min	26	1,011	711	−0.37 (-0.56,0.18)	0.0002	RE	57.36	25	62%	<0.0001
WASO min	13	277	206	0.57 (0.11,1.02)	0.01	RE	56.73	12	81%	<0.00001
SE%	26	1,101	711	−0.61 (−0.80,0.41)	0.0001	RE	56.81	25	61%	<0.0001
SL min	16	454	259	0.34 (0.18,0.50)	0.0001	FE	13.84	15	21%	0.24
SS (event/h)	4	86	83	0.64 (0.33,0.96)	0.0001	FE	1.08	3	0%	0.78
AWN (event/h)	3	59	51	0.72 (0.33,1.12)	0.0003	FE	3.27	2	39%	0.20
N1%	22	805	567	0.37 (0.15,0.59)	0.001	RE	42.77	21	60%	0.0005
N2%	23	805	567	−0.49 (−0.62, 0.36)	0.00001	FE	32.60	22	48%	0.01
N3%	4	113	92	0.17 (−0.11,0.45)	0.22	FE	1.27	3	0%	0.74
SWS%	20	582	357	−0.15 (−0.38,0.08)	0.19	RE	38.18	19	61%	0.0008
REM%	17	560	353	−0.31 (−0.45,0.16)	0.0001	FE	19.00	16	16%	0.27
REML min	3	62	54	0.78 (0.39,1.16)	0.0001	FE	1.44	2	0%	0.49
AHI (event/h)	6	138	109	0.27 (−0.25,0.80)	1.02	RE	7.79	5	62%	0.05
AI (event/h)	10	214	164	0.61 (0.26,0.95)	0.0005	RE	15.36	9	54%	0.03
PLMI (event/h)	21	789	639	1.01 (0.80,1.23)	0.00001	FE	5.25	20	0%	0.51

%, percentage; Q, Cochran's Q statistic; df, degrees of freedom; WASO,wake time after sleep onset; AHI, apnea hypopnea index; AI, arousal index; PLMI, Periodic limb movement index; REM, rapid eye movement sleep; REML, rapid eye movement sleep latency; SWS, slow wave sleep; SE, sleep efficiency; SL, sleep latency; SS, stage shifts; AWN, awakenings number; SMD, standardized mean difference; TST, total sleep time; RE, random-effects model; FE, fixed-effects model.

Among them, the outcome variable of WASO [SMD = 0.57, 95% *CI*: (0.11, 1.02), *I*^2^ = 81%] showed great heterogeneity. Through the sensitivity analysis, it was found that Ferri's (42) research data have a greater impact on the results, resulting in greater bias, so it was deleted. The result of the meta-analysis after the deletion was SMD = 0.44, 95% *CI*: (0.07, 0.80), *I*^2^ = 64%. We analyzed the large heterogeneity produced by this study and considered that it was caused by poor comparability between the experimental group and the control group.

### Risk of bias

Of the 15 parameters included, we showed no publication bias in any of the studies by funnel plot and Egger's test.

## Discussion

### Summary of findings

This is the first systematic review and meta-analysis examining the altered PSG parameters in patients with RLS to the best of our knowledge. The ability to integrate the results of individual studies with relatively small sample sizes is a significant advantage of this approach and allows for the building of a strong body of evidence to illustrate specific issues. Our research mainly found that TST min, SE%, N2, and REM% were significantly decreased in patients with RLS compared with healthy controls, while WASO min, SL min, SS, AWN, N1%, REML, AI, and PLMI were significantly increased. Additionally, there was no significant difference between N3%, SWS%, and the AHI index. The disturbance of sleep continuity and structure in patients with RLS was the main outcome we found, which further illustrates the changes in sleep structure in patients with RLS.

### Sleep changes in RLS

Restless Legs Syndrome may have a serious negative impact on sleep, considering either the associated sleep cycle limb movements or the apparent sensory symptoms. Although the exact pathophysiology of RLS is largely unknown (12). Autopsy, cerebrospinal fluid, and imaging studies have demonstrated the dysregulation of circadian dynamics in patients with RLS (51). Various previous studies on RLS have revealed a decreased fluorine-18-L-dihydroxyphenylalanine (18F-dopa) uptake in the substantia nigra along with a reduction in the postsynaptic dopaminergic activity, providing insight into the role of dopaminergic dysfunction in RLS (52–54). Previous studies have confirmed the ability of the dopamine system to significantly regulate sleep-wake cycles (55). Dopaminergic dysfunction and increased PLMI have been identified by previous studies (56), which similarly affects sleep in patients with RLS. Based on the fact that PSG changes were mainly focused on quantitative sleep parameters, we further found that objective sleep parameters were disturbed in patients with RLS, which demonstrates that patients with RLS have decreased sleep amounts and poor quality of sleep.

Previous studies have widely concluded that SWS is reduced in patients with RLS (57). The disturbance of SWS can exacerbate the neurodegenerative process (58). Our study did not find a change in SWS min% levels between patients with RLS and healthy controls. We speculate that this may be related to the limited number of studies with small sample sizes, making it difficult to observe a significant association. In patients with severe RLS symptoms, increased sleep latency and decreased sleep efficiency were observed (50), which were consistent with our findings. Previous neuroimaging evidence has confirmed that thalamic abnormalities are related to RLS (31). The hyperpolarization of thalamocortical neurons provides a primary regulation for sleep spindle generation and for the reduction of sensory inputs enabling cortical sleep (46, 59). Increased thalamocortical excitation would therefore be expected to produce both the decreased stage 2 sleep and increased wake time seen in patients with RLS (46), which was consistent with our finding that N2% was significantly decreased. A previous study confirmed that the longer WASO duration was associated with symptom severity in drug-free patients with RLS (49). Compared with the control group, we found that patients with RLS had a higher WASO than the control group. REM sleep could contribute to the maintenance of neuronal homeostasis in the brain (11). A previous study has revealed that a decreased REM sleep may exacerbate neurodegeneration (60), which was consistent with our finding that REM min% was significantly lower in patients with RLS. Various previous studies have found that the prevalence rate of RLS was higher in patients with Parkinson's disease (PD) than in the general population (61, 62). Interestingly, a recent study has also suggested that RLS could be a possible preclinical marker of PD (63). However, whether decreased REM sleep could act as a risk factor for patients with RLS developing PD should be validated in a larger population in future work.

### Limitations

There are some shortcomings in our study, and the sample size should be further expanded to improve the quality of relevant studies. In addition, it is necessary to conduct statistical analysis in combination with non-parametric effect sizes, considering the non-normal distribution of some sleep parameters. Finally, patients with specific diagnoses of different subtypes of RLS should be distinguished and studied separately to explore relevant differences. It is worth noting that the difference in bedtime of each subject in the study may also be a potential source of heterogeneity between studies and affect our overall effect size. These limitations suggest that the results of the study should be interpreted with caution and point out that more research is needed.

## Conclusion

Current meta-analysis shows that polysomnography can determine sleep abnormalities in patients with RLS and healthy controls. Especially in patients with RLS, WASO min, SL min, SS, AWN, N1%, REML, and AI increased. In addition, the changes in REM sleep in patients with RLS may reflect the underlying neuropathology and may be an early sign of the process of neuropathological change.

## Data availability statement

The original contributions presented in the study are included in the article/supplementary material, further inquiries can be directed to the corresponding author.

## Author contributions

CG and ZY: wrote first draft and statistics. PX and TZ: statistics and data collection. HZ: conceptualization, resources, and supervision. All authors approved the submitted version.

## Funding

This work was supported by the Henan Medical Science and Technology Research Program (No. 202102310082) and Henan Province Medical Science and Technology Tackling Provincial Ministry Key Projects (SBGJ202102033).

## Conflict of interest

The authors declare that the research was conducted in the absence of any commercial or financial relationships that could be construed as a potential conflict of interest.

## Publisher's note

All claims expressed in this article are solely those of the authors and do not necessarily represent those of their affiliated organizations, or those of the publisher, the editors and the reviewers. Any product that may be evaluated in this article, or claim that may be made by its manufacturer, is not guaranteed or endorsed by the publisher.

## References

[1] Trenkwalder C Allen R Hogl B Clemens S Patton S Schormair B . Comorbidities, treatment, and pathophysiology in restless legs syndrome. Lancet Neurol. (2018) 17:994–1005. 10.1016/S1474-4422(18)30311-9 30244828

[2] Sun S Liu C Jia Y Wu J Li H Li X . Association between migraine complicated with restless legs syndrome and vitamin D. Front Neurol. (2021) 12:777721. 10.3389/fneur.2021.777721 34867766 PMC8634649

[3] Liu HM Chu M Liu CF Zhang T Gu P. Analysis of serum vitamin D level and related factors in patients with restless legs syndrome. Front Neurol. (2021) 12:782565. 10.3389/fneur.2021.782565 34956064 PMC8695899

[4] Oran M Unsal C Albayrak Y Tulubas F Oguz K Avci O . Possible association between vitamin D deficiency and restless legs syndrome. Neuropsychiatr Dis Treat. (2014) 10:953–8. 10.2147/NDT.S63599 24899811 PMC4039397

[5] Balaban H Yildiz OK Cil G Senturk IA Erselcan T Bolayir E . Serum 25-hydroxyvitamin D levels in restless legs syndrome patients. Sleep Med. (2012) 13:953–7. 10.1016/j.sleep.2012.04.009 22704399

[6] Catoire H Dion PA Xiong L Amari M Gaudet R Girard SL . Restless legs syndrome-associated MEIS1 risk variant influences iron homeostasis. Ann Neurol. (2011) 70:170–5. 10.1002/ana.22435 21710629

[7] Schulte EC Kousi M Tan PL Tilch E Knauf F Lichtner P . Targeted resequencing and systematic *in vivo* functional testing identifies rare variants in MEIS1 as significant contributors to restless legs syndrome. Am J Hum Genet. (2014) 95:85–95. 10.1016/j.ajhg.2014.06.005 24995868 PMC4085638

[8] Winkelmann J Schormair B Lichtner P Ripke S Xiong L Jalilzadeh S . Genome-wide association study of restless legs syndrome identifies common variants in three genomic regions. Nat Genet. (2007) 39:1000–6. 10.1038/ng2099 17637780

[9] Ergun U Say B Ergun SG Percin FE Inan L Kaygisiz S . Genome-wide association and whole exome sequencing studies reveal a novel candidate locus for restless legs syndrome. Eur J Med Genet. (2021) 64:104186. 10.1016/j.ejmg.2021.104186 33662638

[10] Zhang Y Ren R Yang L Zhang H Shi Y Sanford LD . Polysomnographic nighttime features of narcolepsy: a systematic review and meta-analysis. Sleep Med Rev. (2021) 58:101488. 10.1016/j.smrv.2021.101488 33934047

[11] Zhang Y Ren R Sanford LD Yang L Zhou J Tan L . Sleep in Parkinson's disease: a systematic review and meta-analysis of polysomnographic findings. Sleep Med Rev. (2020) 51:101281. 10.1016/j.smrv.2020.101281 32135452

[12] Manconi M Garcia-Borreguero D Schormair B Videnovic A Berger K Ferri R . Restless legs syndrome. Nat Rev Dis Primers. (2021) 7:80. 10.1038/s41572-021-00311-z 34732752

[13] Park HR Kim HR Oh S Seong JK Joo EY. White matter tract-specific alterations in patients with primary restless legs syndrome. Sci Rep. (2021) 11:16116. 10.1038/s41598-021-95238-6 34373482 PMC8352949

[14] Baglioni C Nissen C Schweinoch A Riemann D Spiegelhalder K Berger M . Polysomnographic characteristics of sleep in stroke: a systematic review and meta-analysis. PLoS ONE. (2016) 11:e0148496. 10.1371/journal.pone.0148496 26949966 PMC4780740

[15] Moher D Liberati A Tetzlaff J Altman DG Group P. Preferred reporting items for systematic reviews and meta-analyses: the PRISMA statement. PLoS Med. (2009) 6:e1000097. 10.1371/journal.pmed.1000097 19621072 PMC2707599

[16] Sateia MJ. International classification of sleep disorders-third edition: highlights and modifications. Chest. (2014) 146:1387–94. 10.1378/chest.14-0970 25367475

[17] Allen RP Picchietti D Hening WA Trenkwalder C Walters AS Montplaisi J . Restless legs syndrome: diagnostic criteria, special considerations, and epidemiology. Sleep Med. (2003) 4:101–19. 10.1016/S1389-9457(03)00010-8 14592341

[18] Parrino L Ferri R Zucconi M Fanfulla F. Commentary from the Italian association of sleep medicine on the AASM manual for the scoring of sleep and associated events: for debate and discussion. Sleep Med. (2009) 10:799–808. 10.1016/j.sleep.2009.05.009 19564132

[19] Lo CK Mertz D Loeb M. Newcastle-ottawa scale: comparing reviewers' to authors' assessments. BMC Med Res Methodol. (2014) 14:45. 10.1186/1471-2288-14-45 24690082 PMC4021422

[20] Chen X Liu H Wu Y Xuan K Zhao T Sun Y. Characteristics of sleep architecture in autism spectrum disorders: a meta-analysis based on polysomnographic research. Psychiatry Res. (2021) 296:113677. 10.1016/j.psychres.2020.113677 33385781

[21] Mansourian M Rafie N Khorvash F Hadi A Arab A. Are serum vitamin D, calcium and phosphorous associated with restless leg syndrome? A systematic review and meta-analysis. Sleep Med. (2020) 75:326–34. 10.1016/j.sleep.2020.08.022 32950014

[22] Egger M Davey Smith G Schneider M Minder C. Bias in meta-analysis detected by a simple, graphical test. BMJ. (1997) 315:629–34. 10.1136/bmj.315.7109.629 9310563 PMC2127453

[23] Andrel JA Keith SW Leiby BE. Meta-analysis: A brief introduction. Clin Transl Sci. (2009) 2:374–8. 10.1111/j.1752-8062.2009.00152.x 20443922 PMC5350756

[24] Michaud M Paquet J Lavigne G Desautels A Montplaisir J. Sleep laboratory diagnosis of restless legs syndrome. Eur Neurol. (2002) 48:108–13. 10.1159/000062996 12187001

[25] Garcia-Borreguero D Larrosa O Granizo JJ de la Llave Y Hening WA. Circadian variation in neuroendocrine response to L-dopa in patients with restless legs syndrome. Sleep. (2004) 27:669–73. 15283001

[26] Plazzi G Ferri R Franceschini C Vandi S Detto S Pizza F . Periodic leg movements during sleep in narcoleptic patients with or without restless legs syndrome. J Sleep Res. (2012) 21:155–62. 10.1111/j.1365-2869.2011.00942.x 21827556

[27] Hornyak M Feige B Voderholzer U Riemann D. Spectral analysis of sleep EEG in patients with restless legs syndrome. Clin Neurophysiol. (2005) 116:1265–72. 10.1016/j.clinph.2005.02.004 15978488

[28] Ferri R Gschliesser V Frauscher B Poewe W Hogl B. Periodic leg movements during sleep and periodic limb movement disorder in patients presenting with unexplained insomnia. Clin Neurophysiol. (2009) 120:257–63. 10.1016/j.clinph.2008.11.006 19109055

[29] Boehm G Wetter TC Trenkwalder C. Periodic leg movements in RLS patients as compared to controls: are there differences beyond the PLM index? Sleep Med. (2009) 10:566–71. 10.1016/j.sleep.2008.04.009 18753004

[30] Ferri R Manconi M Plazzi G Bruni O Cosentino FI Ferini-Strambi L . Leg movements during wakefulness in restless legs syndrome: time structure and relationships with periodic leg movements during sleep. Sleep Med. (2012) 13:529–35. 10.1016/j.sleep.2011.08.007 22341907

[31] Cha KS Kim TJ Jun JS Byun JI Sunwoo JS Shin JW . Impaired slow oscillation, sleep spindle, and slow oscillation-spindle coordination in patients with idiopathic restless legs syndrome. Sleep Med. (2020) 66:139–47. 10.1016/j.sleep.2019.09.021 31877505

[32] Byun JI Jung KY Lee GT Kim CK Kim BM. Spontaneous low-frequency cerebral hemodynamics oscillations in restless legs syndrome with periodic limb movements during sleep: a near-infrared spectroscopy study. J Clin Neurol. (2016) 12:107–14. 10.3988/jcn.2016.12.1.107 26754783 PMC4712275

[33] Seidel S Garn H Gall M Kohn B Wiesmeyr C Waser M . Contactless detection of periodic leg movements during sleep: a 3D video pilot study. J Sleep Res. (2020) 29:e12986. 10.1111/jsr.12986 32017288 PMC7540172

[34] Schilling C Schredl M Strobl P Deuschle M. Restless legs syndrome: evidence for nocturnal hypothalamic-pituitary-adrenal system activation. Mov Disord. (2010) 25:1047–52. 10.1002/mds.23026 20535825

[35] De Cock VC Bayard S Yu H Grini M Carlander B Postuma R . Suggested immobilization test for diagnosis of restless legs syndrome in Parkinson's disease. Mov Disord. (2012) 27:743–9. 10.1002/mds.24969 22437899

[36] Dauvilliers Y Chenini S Vialaret J Delaby C Guiraud L Gabelle A . Association between serum hepcidin level and restless legs syndrome. Mov Disord. (2018) 33:618–27. 10.1002/mds.27287 29418021

[37] Wetter TC Collado-Seidel V Oertel H Uhr M Yassouridis A Trenkwalder C. Endocrine rhythms in patients with restless legs syndrome. J Neurol. (2002) 249:146–51. 10.1007/PL00007857 11985379

[38] Saletu B Anderer P Saletu M Hauer C Lindeck-Pozza L Saletu-Zyhlarz G. EEG mapping, psychometric, and polysomnographic studies in restless legs syndrome (RLS) and periodic limb movement disorder (PLMD) patients as compared with normal controls. Sleep Med. (2002) 3(Suppl.) S35–42. 10.1016/S1389-9457(02)00147-8 14592166

[39] Chenini S Rassu AL Barateau L Lopez R Carlander B Guiraud L . Increased blood pressure dipping in restless legs syndrome with rotigotine: a randomized trial. Mov Disord. (2020) 35:2164–73. 10.1002/mds.28224 32875658

[40] Ferri R Zucconi M Manconi M Bruni O Ferini-Strambi L Vandi S . Different periodicity and time structure of leg movements during sleep in narcolepsy/cataplexy and restless legs syndrome. Sleep. (2006) 29:1587–94. 10.1093/sleep/29.12.1587 17252889

[41] Hornyak M Feige B Voderholzer U Philipsen A Riemann D. Polysomnography findings in patients with restless legs syndrome and in healthy controls: a comparative observational study. Sleep. (2007) 30:861–5. 10.1093/sleep/30.7.861 17682656 PMC1978374

[42] Ferri R Manconi M Arico D Sagrada C Zucconi M Bruni O . Acute dopamine-agonist treatment in restless legs syndrome: effects on sleep architecture and NREM sleep instability. Sleep. (2010) 33:793–800. 10.1093/sleep/33.6.793 20550020 PMC2881713

[43] Ferri R DelRosso LM Arico D Zucconi M Ferini-Strambi L Picchietti DL . Leg movement activity during sleep in school-age children and adolescents: a detailed study in normal controls and participants with restless legs syndrome and narcolepsy type 1. Sleep. (2018). 10.1093/sleep/zsy010 29365206

[44] Ferri R Cosentino FI Manconi M Rundo F Bruni O Zucconi M. Increased electroencephalographic high frequencies during the sleep onset period in patients with restless legs syndrome. Sleep. (2014) 37:1375–81. 10.5665/sleep.3934 25083018 PMC4096207

[45] Thireau J Farah C Molinari N Bouilloux F Torreilles L Winkelmann J . MEIS1 variant as a determinant of autonomic imbalance in Restless Legs Syndrome. Sci Rep. (2017) 7:46620. 10.1038/srep46620 28425489 PMC5397858

[46] Allen RP Barker PB Horska A Earley CJ. Thalamic glutamate/glutamine in restless legs syndrome: increased and related to disturbed sleep. Neurology. (2013) 80:2028–34. 10.1212/WNL.0b013e318294b3f6 23624560 PMC3716406

[47] Sieminski M Partinen M. Nocturnal systolic blood pressure is increased in restless legs syndrome. Sleep Breath. (2016) 20:1013–9. 10.1007/s11325-016-1333-0 26993341 PMC5016545

[48] Chenini S Delaby C Rassu AL Barateau L Vialaret J Hirtz C . Hepcidin and ferritin levels in restless legs syndrome: a case-control study. Sci Rep. (2020) 10:11914. 10.1038/s41598-020-68851-0 32681031 PMC7367854

[49] Chenini S Rassu AL Guiraud L Evangelista E Barateau L Lopez R . Blood pressure profile and endothelial function in restless legs syndrome. Sci Rep. (2019) 9:15933. 10.1038/s41598-019-52401-4 31685922 PMC6828664

[50] Montplaisir J Boucher S Poirier G Lavigne G Lapierre O Lesperance P. Clinical, polysomnographic, and genetic characteristics of restless legs syndrome: a study of 133 patients diagnosed with new standard criteria. Mov Disord. (1997) 12:61–5. 10.1002/mds.870120111 8990055

[51] Tuovinen N Stefani A Mitterling T Heidbreder A Frauscher B Gizewski ER . Functional connectivity and topology in patients with restless legs syndrome: a case-control resting-state functional magnetic resonance imaging study. Eur J Neurol. (2021) 28:448–58. 10.1111/ene.14577 33032390 PMC7820983

[52] Michaud M Soucy JP Chabli A Lavigne G Montplaisir J. SPECT imaging of striatal pre- and postsynaptic dopaminergic status in restless legs syndrome with periodic leg movements in sleep. J Neurol. (2002) 249:164–70. 10.1007/PL00007859 11985381

[53] Turjanski N Lees AJ Brooks DJ. Striatal dopaminergic function in restless legs syndrome: 18F-dopa and 11C-raclopride PET studies. Neurology. (1999) 52:932–7. 10.1212/WNL.52.5.932 10102408

[54] Ruottinen HM Partinen M Hublin C Bergman J Haaparanta M Solin O . An FDOPA PET study in patients with periodic limb movement disorder and restless legs syndrome. Neurology. (2000) 54:502–4. 10.1212/WNL.54.2.502 10668725

[55] Zhang Y Ren R Yang L Sanford LD Tang X. Polysomnographically measured sleep changes in idiopathic REM sleep behavior disorder: a systematic review and meta-analysis. Sleep Med Rev. (2020) 54:101362. 10.1016/j.smrv.2020.101362 32739826

[56] Fantini ML Michaud M Gosselin N Lavigne G Montplaisir J. Periodic leg movements in REM sleep behavior disorder and related autonomic and EEG activation. Neurology. (2002) 59:1889–94. 10.1212/01.WNL.0000038348.94399.F6 12499479

[57] Leger D Debellemaniere E Rabat A Bayon V Benchenane K Chennaoui M. Slow-wave sleep: from the cell to the clinic. Sleep Med Rev. (2018) 41:113–32. 10.1016/j.smrv.2018.01.008 29490885

[58] Xie L Kang H Xu Q Chen MJ Liao Y Thiyagarajan M . Sleep drives metabolite clearance from the adult brain. Science. (2013) 342:373–7. 10.1126/science.1241224 24136970 PMC3880190

[59] Coulon P Budde T Pape HC. The sleep relay–the role of the thalamus in central and decentral sleep regulation. Pflugers Arch. (2012) 463:53–71. 10.1007/s00424-011-1014-6 21912835

[60] Chauhan AK Mallick BN. Association between autophagy and rapid eye movement sleep loss-associated neurodegenerative and patho-physio-behavioral changes. Sleep Med. (2019) 63:29–37. 10.1016/j.sleep.2019.04.019 31605901

[61] Huang YX Zhang QL Huang CL Wu WQ Sun JW. Association of decreased serum BDNF with restless legs syndrome in Parkinson's disease patients. Front Neurol. (2021) 12:734570. 10.3389/fneur.2021.734570 34764928 PMC8576391

[62] Bhalsing K Suresh K Muthane UB Pal PK. Prevalence and profile of Restless Legs Syndrome in Parkinson's disease and other neurodegenerative disorders: a case-control study. Parkinsonism Relat Disord. (2013) 19:426–30. 10.1016/j.parkreldis.2012.12.005 23333538

[63] Trenkwalder C Allen R Hogl B Paulus W Winkelmann J. Restless legs syndrome associated with major diseases: a systematic review and new concept. Neurology. (2016) 86:1336–43. 10.1212/WNL.0000000000002542 26944272 PMC4826337

